# Leslie’s Reguline Gold

**Published:** 1863-03

**Authors:** 


### LESLIE'S REGULINE GOLD.

This is a very excellent gold foil, prepared by Mr. Leslie of this city, than which we have scarcely found any better, in the properties requisite to make fine and permanent fillings. We have used it considerably for the last few months, and thus far have found it entirely uniform. It deserves to rank as a first class foil. T.

